# The perceptual average in ensemble representation: Neither perceptual nor an average

**DOI:** 10.3758/s13414-025-03187-3

**Published:** 2025-11-26

**Authors:** Jacob Zepp, Chad Dubé

**Affiliations:** 1 Department of Psychology, University of South Florida, 4202 E Fowler Ave, 33620 Tampa, FL United States; 2 Department of Psychology, University of Zürich, Binzmühlestrasse 14, CH-8050 Zürich, Switzerland

**Keywords:** Ensemble Coding, Summary perception, Short-term memory, Cognitive modeling

## Abstract

This report targets the claim that gist representations of visual stimuli, called “ensemble averages”, are perceptual representations of statistics pertaining to stimuli. We report predictions of a mathematical model based on classical memory architectures which assumes ensemble averages are statistical approximations to stimuli, and that those approximations are constructed within short-term memory. We report results of three new experiments that test those predictions. The results support the memory model and contradict the view that representations of ensemble averages are computed early in perceptual processing via parallel processing or neural pooling, suggesting instead that they are computed via control processes acting on item representations held in visual short-term memory. We conclude that the flight toward new mechanisms that has occurred within the ensemble representation literature is ill-advised, and suggest that one first carefully consider what well-established memory models can accomplish in the ensemble “perception” domain.

## Introduction

Intriguingly, recent evidence suggests that gist-like processing, long a focal point in studies of verbal long-term memory, may also support cognitive operations at smaller timescales and in domains other than memory. In particular, the domain of visual perception has produced an ever-increasing wealth of information regarding what appear to be visuo-perceptual analogues of such compact representations. The analogues have many names, such as “ensemble representations” or “summary statistical representations.”

As the terminology indicates, ensemble representations are thought to be “statistical” in nature, in the sense of *representing the statistical properties of stimuli*. Notice that this characterization is distinctly different from the one typically embraced by long-term memory (LTM) theorists, i.e. that memory processes depend upon *statistical approximations to the physical stimulus* (Shiffrin and Steyvers, 1997). Examples of the latter include the error-prone, probabilistic vectors of feature values which are grouped and aggregated in various ways during retrieval in global matching models. While the former characterization (rarely taken up by LTM theorists) provides a conceptual link between ensemble processing and statistical learning, the latter (which is rarely taken up in the ensemble processing literature) provides a conceptual link between ensemble processing and memory theory.

The idea that the visual system represents the statistics of homogeneous ensembles has spawned a host of conceptual models. These include extraction of a mean from the pooled signals of neurons in early visual cortical areas such as V1 (Haberman and Whitney, 2012) or V4 (Utochkin and Tiurina, 2014), a linear amplifier model with no memory system (Baek and Chong, 2020), and recurrent feedback loops in which ensemble representations are computed prior to working memory (Corbett et al., 2023). Proposals involving subsampling of a small number of items have also been advanced, but those models, like their precursors, fail to specify processing mechanisms and are therefore of questionable usefulness in determining the processes involved in ensemble responses.

Our work (Tong and Dubé, 2022; Tong and Dubé, 2022b; Tong et al., 2019; Zepp et al., 2021) has led us to a very different picture, one in which the production of a response in ensemble tasks reflects the activity of control processes (Atkinson and Shiffrin, 1968) that operate on individual item representations held in short-term memory. The idea that ensembles are computed relatively late, on the basis of memory processes, has found recent support in the signal-detection model advanced by Robinson and Brady (2023), though, like all signal-detection models, no cognitive process is actually specified. We propose a memory-based account of mean estimation performance that overcomes this limitation.

In what follows, we explain how we came to view ensemble representation as an STM process (Atkinson and Shiffrin, 1968). We discuss theoretical developments in our lab that culminate in a Non-Selective Transfer Model (NTM). The model, as we will show, includes detail at the level of information processing that is derived from classical memory architectures. The model predictions are held up to test in three new experiments using a mean estimation task. We show how the results allow one to translate between item and resource limits in STM, and reveal a precise quantitative relation between resource and slot limits in our STM data. This latter, unexpected result illustrates the advantages of extensively benchmarked, classical memory-systems approaches over and above the search for new mechanisms.

## Fidelity-based integration and non-selective transfer

Ten years ago, Dubé and Sekuler (2015) reviewed the foundational literature on ensemble perception. In a typical study, participants are shown a display of stimuli varying on some single feature dimension and subsequently they are probed for an estimate of the mean of the feature value that varied in the set. For instance, a display of circles differing in diameter might be presented, followed by a probe stimulus. The participant must indicate whether the probe’s diameter is larger or smaller than the mean diameter of the circles (Ariely, 2001; Chong and Treisman, 2003). Other variants include 2AFC (which probe is larger than the mean) and reproduction, a.k.a. memory recall, tasks (adjust the circle to match your memory of the average size). We concluded at that time that participants compute the statistics of such arrays of stimuli automatically and early in processing, and maintain memory representations of the statistic. In fact, this view remains consistent with predominant thinking in the current literature (see also Corbett et al., 2023). We also reviewed many studies that showed an influence of prior stimulus statistics on single-item judgments which have no nominal connection to explicit averaging tasks. These patterns led us to conclude that memory representations of statistics are computed obligatorily. As of this writing, it appears that part of what we concluded was correct and part of it was not.

Specifically, the current literature does seem to support the notion that something is in fact encoded and remembered obligatorily that may contribute both to explicit and implicit ensemble effects. However, work from our own lab over the past decade has led us to seriously question the conclusions that i) participants form representations of statistics and ii) that such representations are formed early on in sensory processing, globally, in parallel, outside of or prior to attentional selection, via neural pooling in visual cortex, etc.

In Tong et al. (2019) the authors asked whether, as several models within Ensemble Theory claim, all stimuli contribute in roughly equal measure to ensemble computations (“global pooling”). Since, as detailed by the authors, such models have been used to explain ensemble judgments for both simultaneous and sequential-presentation of items in ensembles, Tong et al. presented participants with simple unidimensional stimuli presented in a sequence. In some experiments line lengths were used, in another one numerals were used. On each trial, participants were tasked with estimating the rolling mean using a recall/reproduction probe stimulus. In all experiments, the distribution from which stimulus feature values were drawn was shifted midway through the experimental trials. The authors showed how, in the typical design, ideal observers taking a weighted average, an equally-weighted arithmetic average, and a single item subsample, are indistinguishable. However, adding a distribution shift strongly differentiates the three models’ predictions in the wake of the shift. The results showed a clear pattern, consistent across all experiments and observers: Participants’ responses at the trial-by-trial level fell between the post-shift predictions based on the single most recent stimulus and those based on an equally-weighted average, instead falling within range of a recency-weighted ideal observer.

The Tong et al. (2019) data were also consistent with a variable precision account of item representations used in the task, since the best-fitting models assumed there is no clear discrete cutoff for how many items were used, but rather a continuously-varying weight which drops to some $\varepsilon > 0$ over increasingly-previous items.

In their Experiment 4, the authors obtained results from an experiment in which participants viewed blocks of short sequences of stimuli and made a single mean estimate for the sequence at the end of each one. The parameter values of the recency model from prior experiments provided highly-accurate predictions for the results, which included a set size (sequence length) variable not present in the earlier experiments. Taken as a whole, the results provided some initial indications that, at least for sequential averaging tasks, memory representations varying in their fidelity were combined by giving higher weight to more detailed, less uncertain memory representations (i.e., those that would fall on the recency portion of a recall curve, long linked to STM storage).

A subsequent paper by Tong and Dubé (2022) provided further clues regarding memory systems, and addressed whether our results would generalize to simultaneously-presented stimuli, like the arrays typically used in foundational ensemble perception studies. First, the authors examined variants of the design with a single estimate following sequences of varying lengths. Results were consistent with their prior findings, but also revealed an influence of the global mean over prior stimuli on current mean judgments (see also Whiting and Oriet, 2011). The authors included single-item sets as well, and demonstrated that errors increase in a nonlinear way, with a large increase from 1 item to 5 item sequences, then little difference between 5 and 10-item ones. In two experiments, the $R^2$ relating participants’ estimates to the true means showed a nonlinear drop with set size, with an “elbow” around 4 items, consistent with estimates of the “slot” capacity within visual short-term memory (vSTM) models that include slots, and prior estimates for item limits (Cowan, 2001). A simultaneous array variant of the experiments replicated the global central tendency effect and also showed weighting patterns that were obtained via ideal observer modeling and were consistent with fidelity-based weight-assignment in computing a mean: those items closest to the center of the display tended to have the greatest weight.

Tong and Dubé (2022) formulated a computational modeling framework called the Fidelity-Based Integration model (FIM). The model takes on a variable precision picture of vSTM in which items are represented as distributions of certainty or likelihood over a feature-value axis for the relevant dimension of judgment. The system samples feature values from an entire set of such distributions, with the spread of the distributions (i.e. their fidelity) determining the number of samples taken from each particular distribution. The samples are then combined into a single distribution, from which a final sample is taken for response. Within this simple framework, which incorporates several of the more successful ideas from prior models, a variety of tasks can be instantiated including single-item estimation and explicit averaging. The authors were able to successfully capture all of the patterns in their data with the FIM.

In a follow-up paper, Tong and Dubé (2022b) used the FIM to provide a unifying account of two seemingly disparate phenomena in the domain of single item estimation: serial dependency and central tendency bias. Four established findings in single-item estimation were of particular interest: i) central tendency bias, ii) nonzero and recency-prioritizing regression weights on prior nontarget stimuli, iii) positive slopes relating estimation error to relative feature value of prior trial, and iv) the decrease in that slope with lag. Of a number of alternative accounts, only FIM was able to account for all of these patterns. Two novel predictions were then derived: v) relative fidelity of targets and non-targets will determine which item(s) carry more weight, in the direction of the item with higher fidelity and vi) biasing effects of recent non-target trials will be greater than the biasing effects of central tendency. New single-item estimation experiments were reported to test these predictions, derived solely from FIM simulations, and the predictions were clearly confirmed.

In sum, FIM contradicted Ensemble Theory because its predictions were derived from the assumption of integration by fidelity, so that only some of the items from stimulus sets had any significant impact on computation of an ensemble average. The averages estimated by participants, then, could be accounted for by an integration process that may not represent an “average” at all.

Although verbally described as a vSTM process, the FIM was intended to be a general, broad *algorithmic* framework within which to instantiate specific *mathematical* models. In one sense that is a strength of FIM, but in another sense it is also a weakness. Specifically, there is nothing in the FIM equations that clearly links it to particular memory systems. Therefore, we must ask the question of what system or systems are involved in the integration process.

To answer this question, Zepp et al. (2021) took a different approach, by using an Atkinson-Shiffrin framework to direct their empirical work. They argued that, if as the perceptual view states participants encode mean representations prior to filtering into vSTM, then one should in principle be able to demonstrate dependence of ensemble coding on visual sensory storage mechanisms.

In this domain of iconic memory research, two main processes, distinct from afterimages, have been identified: visual persistence and informational persistence. Visual persistence can be measured via the classic successive-field task (Eriksen and Collins, 1967; Hogben and di Lollo, 1974; Wutz et al., 2016). In such tasks, participants must make a judgment about stimuli in arrays that are split across two successive frames, with a very short and variable inter-frame duration between them. Some judgments are impossible unless participants combine information across the two frames, forming an integrated representation. These are called “integration trials.” Other judgments are impossible unless participants do the opposite, i.e. differentiate between the frames. These are called “segregation trials.” Variation in the inter-frame interval duration (IFI) produces a crossover in performance, with integration decreasing linearly with IFI and segregation increasing.

To this well-established paradigm Zepp et al. (2021) added a third task, which was to estimate the mean orientation of the stimuli (Landolt Cs). Importantly, the only thing differing across the integration, segregation, and averaging trials was the judgment required of participants. Even the reproduction probe was included in the probe arrays of all trials, and probe arrays also doubled as pattern masks on all trials. The results showed that precision in mean estimation was high and constant across all IFIs, showing no dependence on temporal integration mechanisms. Turning to informational persistence, Zepp et al. found that statistics from prior studies of transfer rate predicted two items were filtered into vSTM during the tasks. Then, the authors compared 12 different observer models with zero free parameters in fits to the data broken down by the intra-frame orientation variance, which was varied parametrically in the study. Of these 12 models, only one provided an adequate prediction of the data. That model assumed that participants used two items to derive their estimates of mean orientations, in agreement with the predictions from transfer rate and time parameters of the study.

The authors concluded that participants compute ensemble means using the contents of vSTM, at a point much later in processing than a perceptual ensemble theory allows. They concluded that those contents are the result of non-selective readout of a relatively small amount of information from iconic memory, in contrast to both the perceptual account *and* subsampling accounts of ensemble representation.

What all of these findings suggest is that, in contrast to the perceptual view which we call Ensemble Theory, a significant range of data that has been interpreted as reflecting early sensory and *perceptual* processes such as neural pooling, population coding, pre-attentional processing, parallel processing, etc. can in fact be handled via established *memory* processes and models.

With a memory-systems perspective on ensemble coding we can begin to investigate relevant information processing characteristics, such as the quality and quantity of information available to make ensemble responses. Transfer of information into the manipulable and durable vSTM is subject to bottlenecks on both the information transfer rate and the number of items available for report. These transfer dynamics have been well described in the literature through modeling of whole and partial-report response data. From the memory-systems perspective, encoding within the ensemble task should be similar to that of the whole-report procedure, in that both tasks require subjects to transfer as much information from a display as possible into vSTM to make a response. In either task, no specific item is cued and therefore the contents of vSTM are a product of naturalistic and passive encoding biases, a process Gegenfurtner and Sperling (1993) labeled *non-selective transfer*, following Averbach and Coriell (1961).

In this work, we report three experiments that test the memory-systems notion further. In Experiment 1, we use a whole-report task with oriented elements to predict the same participants’ estimates of mean orientations on matched trials. In Experiment 2, we parametrically vary the range of feature values within the ensembles as well as ensemble cardinality. In Experiment 3, we use an orientation pattern mask to control readout from iconic memory over several levels of readout time, and compare this with a no-mask condition. We formalize a Non-Selective Transfer Model (NTM), which translates the Gegenfurtner and Sperling (1993) readout equations into fidelity-based precision equations, to predict mean estimation precision as a function of encoding time for the arrays in these experiments. Those predictions were verified. We were also able to reverse the process: using observed ensemble precision values, we were able to predict average vSTM representational precision and the number of items sampled into vSTM. The findings and conclusions contradict Ensemble Theory and support a memory-systems model instead.

**Fig. 1:**
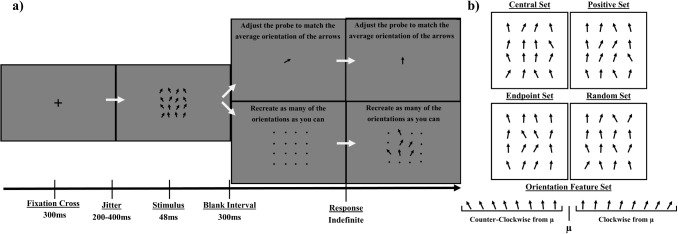
An example of the Experiment 1 trial progression and stimuli feature x location configurations. Panel a: The task (orientation averaging vs whole-report) and related response varied by block. Stimuli were equated between the blocked tasks. Panel b: An example of the four spatial configurations of the oriented elements. A sample feature distribution, centered around a mean of $0^{\circ }$ is included. The central set structures the center of the display tightly around the frame mean, with low range. The positive set biases the center of the display away from the frame mean, with low range. The endpoint set makes the center of the display have the lowest feature homogeneity, but non-biased. The random set control is most similar to general ensemble experiments

## Experiment 1: Relating whole-report and ensembles

Following previously described work from our lab, ensemble reports are expected to inherit biases present within the whole-report task, such as a bias towards features that are presented closer to foveation. To make these biases detectable, a set of stimulus feature x location configurations are generated (Fig. 1b), with overall feature range also varying between a high and low level. The underlying feature distribution is preserved in each configuration, with only locations of defining features being manipulated. The same set of stimuli is used between the two tasks, with only the response varying.

## Methods

### Participants

Twenty University of South Florida undergraduate students were recruited from the university participant pool, ranging in age from 18-26 years old (mean age = 19.15 years, SD = 1.95 years.) Twelve participants were female, six were male, and two were non-binary. All had normal or corrected-to-normal vision. Participants completed the experiment in one session and received course credit as compensation for their time. No participant within this experiment participated in Experiments 2 or 3, nor did they have any prior knowledge of the experiment beyond the provided instructions. All experimental procedures were approved by the University of South Florida IRB.

### Apparatus and stimuli

Stimuli were created using the Matlab Psychophysics Toolbox (Brainard, 1997; Pelli, 1997; Kleiner et al., 2007), and were presented on a 1920 $\times$ 1080 pixel resolution 25” LCD monitor with a 240 Hz refresh rate. The monitor was tied to a PC that was custom built to optimize timing and processing speed, and to minimize lags. The PC’s frame rate was 125 fps, and was benchmarked in FRAPS (a computer monitor frame rate benchmarking software; Beepa, 2013) during test runs of the experiment to verify observed frame durations matched those specified in Matlab. Participants were seated approximately 100 cm from the screen, making each pixel of the screen approximately $0.02^{\circ }$ of visual angle.

The stimulus set consisted of sixteen black arrows, presented against a uniform gray background (RGB: [127 127 127]). Each arrow varied monotonically along the orientation feature dimension, bounded between [$-90^{\circ }$, $90^{\circ }$], with $0^{\circ }$ representing vertical “up”. The arrows were organized into an invisible 4 $\times$ 4 grid of equally-spaced possible presentation locations, positioned about the center of the screen. Each arrow was presented within an invisible box measuring $0.5^{\circ }$ of visual angle. Individual positions of arrows within each grid square were slightly jittered (randomly and independently selected from a normal distribution with $\sigma =3$, truncated between ± 30 pixels for both x and y axes) on each trial. The invisible stimulus grid was $3.5^{\circ }$ of visual angle in horizontal and vertical size, with $0.5^{\circ }$ of space between each individual grid element location, measured center to center.

Orientation feature values for each arrow within a display were uniformly spaced around a set mean value, with a set range value. One of four unique orientation mean values ($\mu$ = $-60^{\circ }$, $-30^{\circ }$, $30^{\circ }$, $60^{\circ }$) were chosen for each display, around which the 16 orientation feature values in a display were spaced. The chosen trial mean value was never a feature value within the current display. The spread of orientation feature values varied between a narrow range ($\mu \pm 15^{\circ }$) and a broad range ($\mu \pm 30^{\circ }$). The combination of mean and range values gives 8 unique feature distributions (4$\mu$ $\times$ 2 ranges).

The orientation values of arrows within the central four elements of the 4x4 stimulus grid on a particular trial were structured to fit into one of four different configurations (see Fig. 1b):

- Central set: The central four elements were selected to be the four values closest to the frame mean, such that the central items are low in spread and centered around the frame mean.
- Positive set: The central four elements were selected to be the four values with the furthest positive distance from the frame mean, such that the central items are low in spread and systematically larger than the frame mean.
- Endpoint set: The central four elements were selected to be two values from each end of the feature distribution, such that the central items are high in spread and centered around the frame mean. The non-central items therefore have greater homogeneity than the central items.
- Random set: The central four elements were randomly sampled from the set of sixteen feature values in the feature distribution, as is standard within ensemble averaging experiments.

Each orientation feature value was selected without replacement from the feature distribution. Therefore, structuring the central location feature values also constrains the surrounding non-central values (e.g. if the central four features were chosen to be closest to the frame mean, the surrounding twelve locations would contain items farther from the frame mean; see Fig. 1b).

The set of 4 mean values, 2 range values, and 4 structural configurations provided 32 unique stimulus frames per participant. Each unique frame was repeated 7 times, in a randomized order within a block, for 224 trial frames per block. The same set of stimulus frames was used within (but not between) each pair of averaging and whole-report task blocks. The presentation order was randomized between the first and second pair of task blocks (i.e. between the first and second orientation/whole-report pair).

Outside of the constraints imposed by the manipulations, spatial locations of individual orientations were randomly allocated within an ensemble, from one trial to the next, and from one participant to the next.

**Fig. 2:**
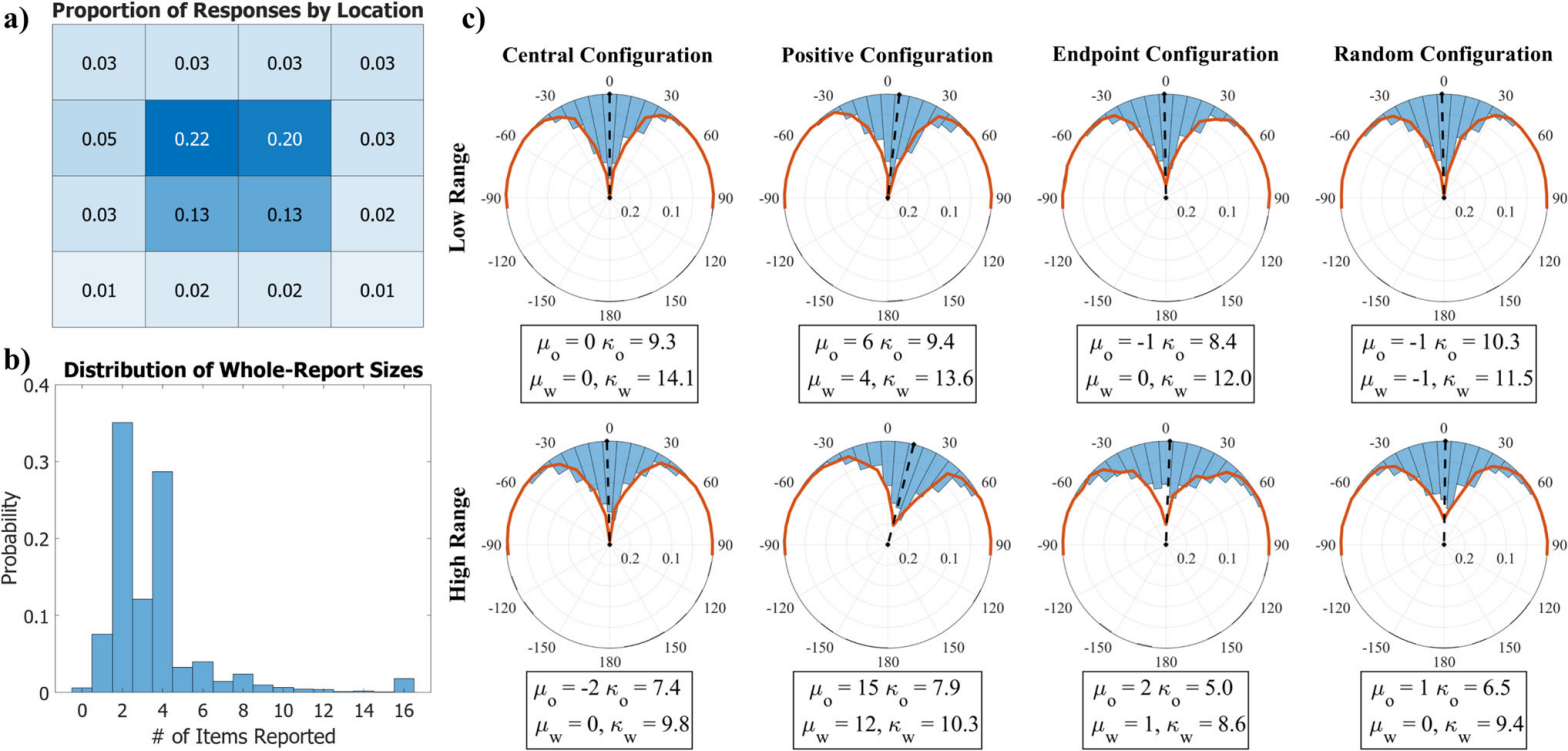
Results of Experiment 1. Panel a: Heatmap describing the distribution of items reported from each possible item location within the whole-report task. Panel b: Distribution of whole-report sizes. Participants were asked to respond with as many items as they could remember, out of 16 items. The number of items reported could range from no items (0) to all items (16). Panel c: Circular error histograms displaying participant orientation averaging error distributions for each condition. Longer blue bars indicate more concentration around a bin. Dashed black lines represent the average participant error value of each condition. Red lines display a fit of whole-report response averages to orientation averaging data. The distribution of ensemble task responses are shown with the blue histogram bars. Mean error and precision parameter estimates from the participant ensemble data are listed with $\mu _o$ and $\kappa _o$. Whole-report model fits parameter estimates are listed with $\mu _w$ and $\kappa _w$

### Procedure

Before the first block of experimental trials, a series of practice trials was given to familiarize participants with each task. A still image displaying the trial progression (similar to Fig. 1a) was shown. participants were instructed that each arrow displayed would be pointed some degree of “upwards” between horizontal left ($-90^{\circ }$) and right ($90^{\circ }$), to orient participants on the directionality of the stimuli. The practice trials began with a slow presentation of each display, which progressed in speed until reaching the same speed as the experimental trials. Upon completion of the practice trials, the experimental trials immediately followed.

Each trial initiated with a central fixation cross, displayed for 300ms. After a randomly jittered interval (between 200-400ms), the stimulus frame was displayed briefly for 48ms. A 300ms post-stimulus delay was given before participants were prompted for a response.

Within the orientation averaging task blocks, the response screen contained a single arrow in the center of the display. The response arrow probe was initialized to a random orientation value, sampled from a uniform distribution between [$-180^{\circ }$, $180^{\circ }$]. Participants moved the mouse up or down to rotate the response arrow probe until it matched what they believed was the average orientation of the previously displayed set of 16 arrows (Fig. 1a). The range of responses was not restricted, such that the response space allowed for any orientation between [$-180^{\circ }$, $180^{\circ }$]. Once the probe was appropriately oriented, participants locked in responses by pressing the space bar.

In the whole-report task blocks, a 4x4 grid of black dots was initially displayed on the response screen. Each dot occupied a matching location of an arrow that was displayed on the stimulus frame. If a participant remembered a specific arrow’s orientation from a location, the participant clicked the mouse on the dot in the location of the remembered item. Clicking on the dot made an arrow (with initial orientation $0^{\circ }$) appear in the selected location. The participant recreated the orientation of the selected item by holding down the mouse button and moving the mouse up and down to rotate the selected arrow. This process was repeated for as many items as a participant could remember (Fig. 1a), after which the participant pressed the space bar to lock in a response. The orientation value of all item responses, as well as the location and order of responses were recorded.

In both tasks, participants were allotted an unlimited response time though instruction to respond as quickly and accurately as possible was given. Progression to the next trial occurred at the press of the space bar in both tasks.

## Results

### Whole-report task

Figure 2a displays the proportion of reports from each possible stimulus location in the display. As was the case in Tong and Dubé (2022) for line lengths, participants were overwhelmingly likely to report items from the center of the display (68% of responses). A particular bias was present to report items from the upper two central locations (42% of responses). Report location behavior was not significantly altered by changes in the stimulus configuration or feature distribution range conditions.

Participants were most likely to report between 2 and 4 items within each whole-report trial (Fig. 2b). As the whole-report procedure measures non-selective transfer from iconic memory to vSTM for reports, the concentration of report sizes described here is well within information transfer bottlenecks and vSTM capacity estimates (Sperling, 1960). This distribution of whole-report sizes was not affected by changes in range or stimulus configurations.

Within this experiment, the accuracy of a participant’s whole-report response within a specific location was not a key metric of interest. Instead, the focus was primarily on the underlying encoding dynamics in a nonselective task, with a hope to infer the dynamics occurring during the ensemble averaging task. However, it is of interest to point out the presence of central tendency biasing in the responses to the whole report items. Our group has previously presented the FIM framework, which describes the relationship between ensemble coding and central tendency biasing (Tong and Dubé , 2022b) and therefore we will not divert from our main thesis by restating those thoughts here again.

### Orientation averaging task

Participants’ orientation averaging performance was analyzed using a Bayesian linear mixed-effects distributional regression. Response error was expressed in radians for the analysis. Fixed effects for each condition, as well as all interactions, were modeled with random slopes and intercepts for estimates of the Von Mises distribution mean and precision terms. The comparative intercept condition was the low range x random configuration condition. Weakly informative normal priors [$\mathcal {N}(\mu = 0,\,\sigma = 30)$] were set on all parameters.

The Bayesian regression analysis was conducted using the BRMS package (Bürkner, 2018, 2017) in R (R Core Team, 2021), with 4 chains of 10,000 samples each. Chains were visually checked for convergence, and $\hat{R}=1$ for all estimated parameters. Parameter estimates with 90% posterior sampling credible intervals that exclude 0 are discussed.

The analysis shows that average orientation ensemble error was significantly positively biased away from the true set mean value when the center of the display also had a positive bias (estimate=0.07, 90% CI:[0.05-0.08]), in comparison to a random location x feature structure. This positive error bias increased in magnitude when the range of the feature distribution was increased (estimate=0.05, 90% CI:[0.03-0.08]). Adjusting the range alone did not lead to significant average ensemble report bias. Similarly, no other location configuration had a significant biasing effect on the ensemble.

A log-link function was used for precision effect parameter estimation, therefore all estimates are expressed on a log-scale. Contrary to global pooling and other “early” models, ensemble precision significantly decreased when the range of the feature distribution increased (estimate=-0.31, 90% CI:[-0.42 - -0.19]). The only feature/location configuration that significantly decreased ensemble precision was the endpoint configuration, when the center of the display was wide in spread (estimate=-0.14, 90% CI:[-0.25 - -0.03]). No other stimulus configuration affected ensemble precision significantly.

Computation of the variance partition coefficient showed significant variation between participants, with 19% (90% CI:[0.05-0.32]) of data variance being accounted for by random effects clustering of participant individual differences.

### Non-selective transfer model

One hypothesis for the process of ensemble generation is that summaries are generated over the set of noisy features that have been encoded into vSTM (Zepp et al., 2021). In an ensemble coding task, the instruction is generally to remember all items such that no selective priority should exist for the transfer of one item over another. Sampling is therefore non-selective from iconic memory to vSTM within an ensemble memory task. This creates a link between the whole-report task (in which participants report the non-selectively transferred items) and the ensemble task (in which participants report a summary value over the non-selectively transferred items).

**Fig. 3:**
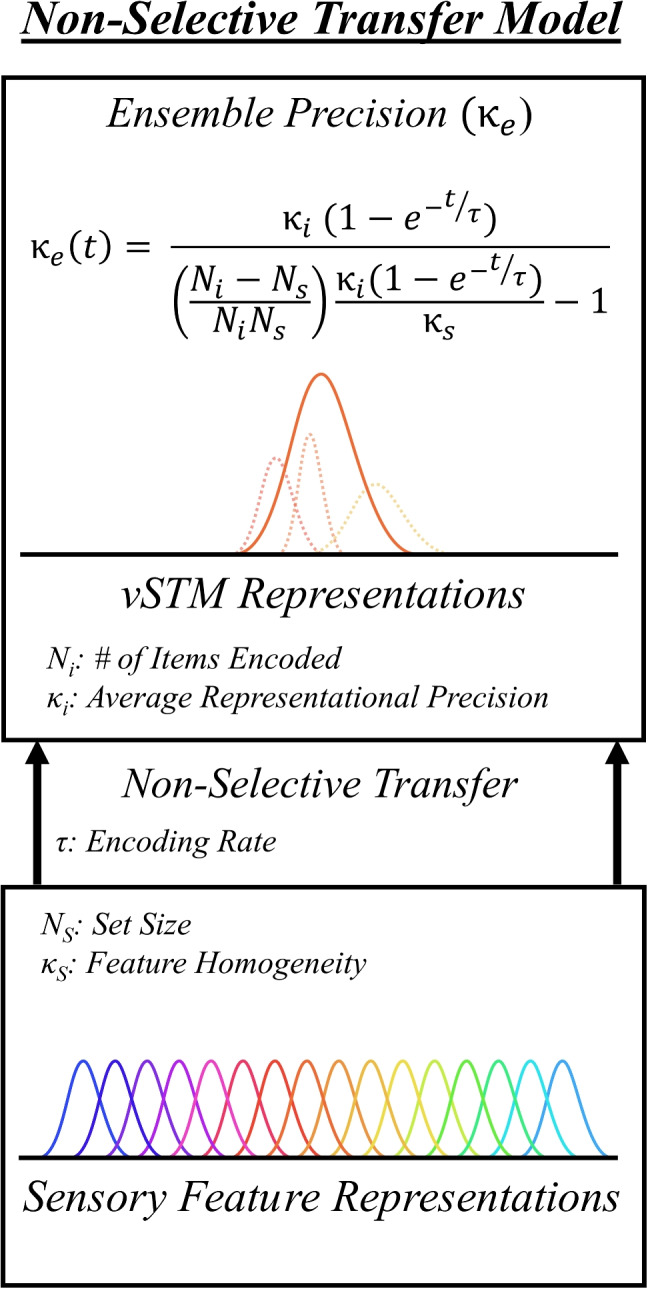
Non-selective transfer model of ensemble averaging. The ensemble code is a representation generated over item information encoded into vSTM. The set of integrable items within vSTM is dependent on sensory representations and the encoding process. Generally, encoding during the ensemble task is not directed, though can be biased through attentional effects. Sensory factors (such as foveal biases) will passively bias selection towards the center unless controlled for

A representation of ensemble coding based on non-selective transfer is shown in Fig. 3. A sensory representation of all items in a scene is present in iconic memory. Items are sampled without replacement into vSTM through a non-selective transfer function. The link between the whole-report and ensemble tasks allow for the estimation of the transfer function, based on the structure of the whole-report responses. Within vSTM, transferred items are represented with some level of noise. In a whole-report task, the items from vSTM are reported. In an ensemble task, a control process acts on the items in vSTM to generate a summary representation, which is then reported.

A fit of the model can be seen in Fig. 2c. The fit was generated by taking the average over each set of items reported in the whole-report task. Doing so mimics the control process present in Fig. 3. This model has zero free parameters, as the response is generated through the participant’s own responses on a matched task.

The NTM modeling, solely via whole-report responses, predicted the ensemble averaging data quite well. The main effects of a positive bias in the positive stimulus configuration and low precision in the endpoint configuration, both of which become more pronounced with increasing range, are present in the whole-report model fits. The fit of this model, with zero free parameters, provides credibility to the underlying theory of ensembles generated from items following their transfer into vSTM.

**Fig. 4:**
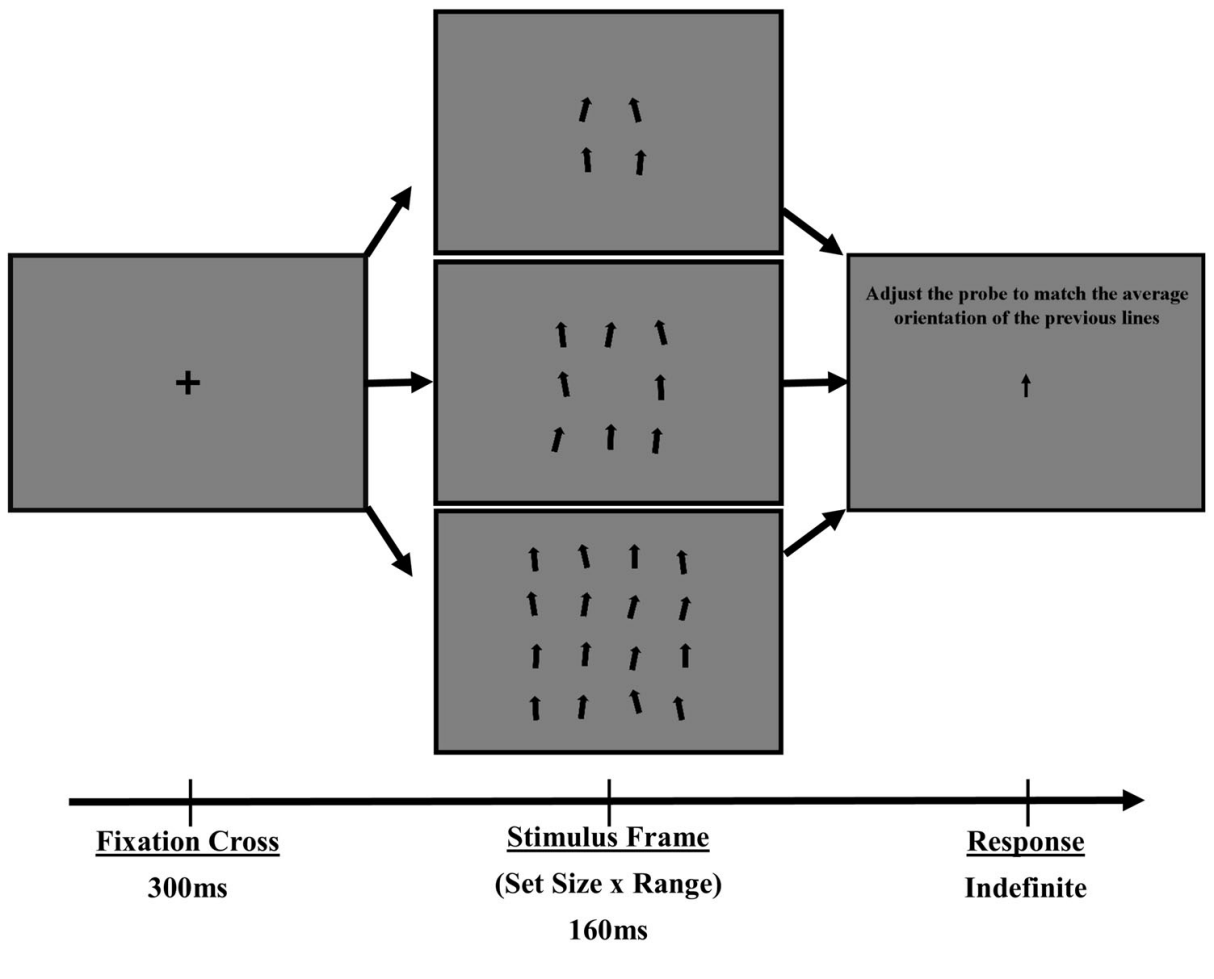
An example of trial progression for Experiment 2. Participants completed all conditions. Set size was blocked, but range varied within blocks

The validation of the model’s core assumptions of a vSTM based summary generation leads to a formalization of the relation between stimulus characteristics, representational characteristics, and the precision of the ensemble within the NTM:

1 $$\begin{aligned} \kappa _e = \dfrac{\kappa _i}{\left( \dfrac{N_s - N_i}{N_s N_i}\right) \left( \dfrac{\kappa _i}{\kappa _s}\right) +1} \end{aligned}$$

Where $\kappa _e$ is the ensemble response precision, $\kappa _i$ is the average individual item representation precision^1^ (i.e. vSTM “resources”), $N_i$ is the average number of items sampled into the ensemble representation (i.e. items available within vSTM), $\kappa _s$ is the precision of the set of feature values within a stimulus, and $N_s$ is the set size of the stimulus ensemble. Using this relation, we can elucidate the relationship between changes in “slot” and “resource” allocations needed to model expected patterns of results. This relationship will be used to guide the following two experiments.

## Experiment 2

This experiment uses manipulations of stimulus distribution characteristics to test the assumptions of NTM. The model expects that sampling behavior should be largely unaffected by changes in stimulus homogeneity, and that decrements in ensemble precision will come from how representative any subsample is of the original population.

## Methods

### Participants

Twenty University of South Florida undergraduate students were recruited for participation in this study. The data from one participant has been excluded from the current results due to a failure to follow experimental instruction. All had normal or corrected-normal vision. Participants completed the experiment in one session and received course credit as compensation for their time. No participant within this experiment participated in Experiments 1 or 2, nor did they have any prior knowledge of the experiment beyond the provided instructions. All experimental procedures were approved by the University of South Florida IRB.

**Fig. 5:**
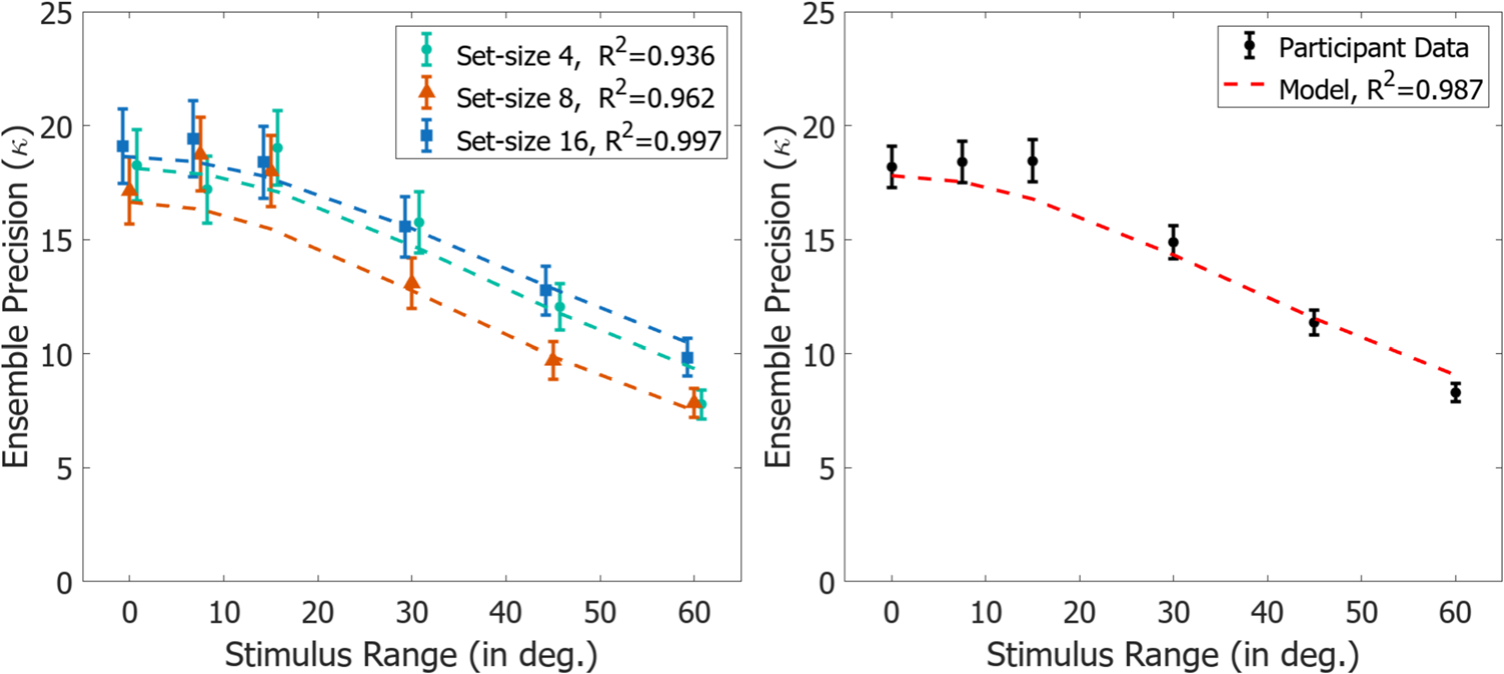
Left panel: Participant error precision from Experiment 2 for each range and set size condition, with associated 95% confidence intervals. Participants showed a decrease in precision with increasing stimulus distribution range, with no statistical effects of set size on performance. Dotted lines represent a fit of Eq. 1 to the participant data with a fixed average subsample size, $N_i$, and fixed item representational precision, $\kappa _i$, for each set size condition. Right panel: Participant data and fits collapsed over set size conditions

### Design

The experiment was organized as a 3 (set size: 4 vs 8 vs 16 items) x 6 (within-ensemble orientation range: $0^{\circ }$, $7.5^{\circ }$, $15^{\circ }$, $30^{\circ }$, $45^{\circ }$, $60^{\circ }$) fully within-participants design. Each participant completed a total of 900 trials and participated in each condition. The experiment was segmented into 3 blocks, one for each set size level, with 300 trials per block. Range varied within each block, giving 50 trials per set size x range condition. An example of the design for Experiment 2 is given in Fig. 4.

### Apparatus and stimuli

Stimulus frames varied in set size, with sizes of 4, 8, or 16 items on a display. For each set size, items were organized into an invisible square pattern around the center of the screen. At set size 4, items were organized in a 2x2 square, at set size 8 items were in a 3x3 grid with the central position unfilled, and at set size 16 the items were in a 4x4 grid. The set of stimuli on a frame occupied a size on the screen of $1.5^{\circ }$, $2.5^{\circ }$, or $3.5^{\circ }$ degrees of visual angle respectively. The stimulus frame was displayed for a duration of 160ms. Stimulus mean feature values varied randomly in the interval [$-60^{\circ }$,$60^{\circ }$], with a range fixed to one of 6 values ($0^{\circ }$, $7.5^{\circ }$, $15^{\circ }$, $30^{\circ }$, $45^{\circ }$, or $60^{\circ }$) per frame. All other properties of the experiment were similar to that of Experiment 1.

### Procedure

Each trial began with a centrally presented fixation cross, displayed for 300ms. After a variable delay, the stimulus frame was presented centrally on the display for 160ms. The response screen was displayed 300ms after stimulus offset. Participants were directed at the response screen to adjust a probe arrow towards the average orientation of the previously displayed items. The probe arrow was initialized to a random starting orientation, from which it could be rotated by moving the mouse up or down. Participants had an unlimited time for response, and were instructed to right-click the mouse once the probe was pointing in the direction of the average orientation. The next trial began after response.

## Results

A Bayesian distributional linear mixed-effects model was used to analyze participant orientation averaging performance. All fixed and random effects, as well as all interactions, were included in the model for estimates of the Von Mises distribution mean and precision terms. Weakly informative normal priors were set on all parameters [$\mathcal {N}(\mu = 0,\,\sigma = 30)$].

The analysis indicated a main effect of stimulus range of response precision (estimate=-0.01, 90% CI:[-0.02 - -0.01]), though not on set size or the interaction. The results are displayed in Fig. 5.

As can be seen in Fig. 5, a non-linear decline in response precision appears to be present with decreasing similarity across all set sizes. This behavior is predicted by Eq. 1, as the stimulus variance ($\kappa _s$) is increasing with increasing range. As no changes are present, beyond changing the range of stimulus variability within each set size, we can assume that the item representational precision ($\kappa _i$) and number of items sampled ($N_i$) remains constant. Support for these assumptions comes from the invariance of whole-report response characteristics (spatial locations where items were most sampled and how many items were sampled) across conditions within Experiment 1. In this interpretation, the spatial filter of item selection will be relatively constant within a certain set size (as the spatial layout of the display items is constant), though will change between set-sizes as the spatial layout of the display changes (Reynolds and Heeger, 2009). The precision within which items are represented is also constant within a set size, though the precision of the ensemble representation relative to the full stimulus feature distribution will decline as any subsample from the feature set becomes a worse estimator of the total population distribution (i.e. as variability in the stimulus feature distribution, $\kappa _s$, increases).

When stimulus feature distribution similarity is high, the precision is dominated by item representational noise and item feature values become less distinguishable. When the stimulus feature distribution is completely homogeneous ($\kappa _s$ = $\infty$), as is the case in the Range = 0 condition, the relation in Eq. 1 collapses to $\kappa _e$=$\kappa _i$. We can therefore use the participant ensemble response precision value when Range=0 as our estimator of $\kappa _i$ in the equation. Given that no manipulation is present within this experiment to limit individual stimulus encoding properties, we make an assumption that the representation precision ($\kappa _i$) will remain constant across range conditions within each set size.

Small changes in stimulus similarity likewise produce small changes in expected response precision from the homogeneous/identical case. At low levels of stimulus feature distributional similarity, the items become more distinct in their contribution to the ensemble representation and it becomes much more tractable to determine the number of items being sampled apart from item and measurement noise. We can therefore look to the higher range conditions to gain an estimate of the final parameter: the number of items sampled, $N_i$. Rearranging Eq. 1 for $N_i$ gives:

2 $$\begin{aligned} N_i = \dfrac{N_s}{\left( \dfrac{\kappa _i - \kappa _e}{\kappa _i \kappa _e}\right) \left( N_s \kappa _s\right) +1}, \end{aligned}$$

with $\kappa _e$ as the ensemble response precision within each condition, $\kappa _i$ being the value of $\kappa _e$ for each set size condition when Range=0, and $\kappa _s$ and $N_s$ being the stimulus distribution precision and set size, respectively. To remove as much measurement noise as possible, we average over the estimates of $N_i$ at the higher range conditions (Range:[30 - 60]) to estimate a stable value of $N_i$ for each set size condition. The derived estimated average number of items sampled were $N_i$=[1.95,1.49,2.27] for set sizes 4, 8, and 16 respectively. The resulting fits of this procedure for each experimental condition, as well as collapsed over set-sizes, is shown in Fig. 5. The provided model fits the participant data exceedingly well, with no free parameters.

## Experiment 3

The assumption that ensembles are generated over information transferred into vSTM is tested. Limitations on encoding time through masking classically limit vSTM information availability. The influence of a rolling accumulation of information (Whiting and Oriet, 2011) is also tested using 2 global distributions of trial means in a block (normal vs bimodal).

## Methods

### Participants

Twenty-five University of South Florida undergraduate students were recruited from the university participant pool, ranging in age from 18-20 years old (mean age = 18.65 years, SD = 0.7 years.) Twelve participants were female, twelve were male, and one was non-binary. All had normal or corrected-to-normal vision. Participants completed the experiment in one session and received course credit as compensation for their time. No participant within this experiment participated in Experiments 1 or 2, nor did they have any prior knowledge of the experiment beyond the provided instructions. All experimental procedures were approved by the University of South Florida IRB.

**Fig. 6:**
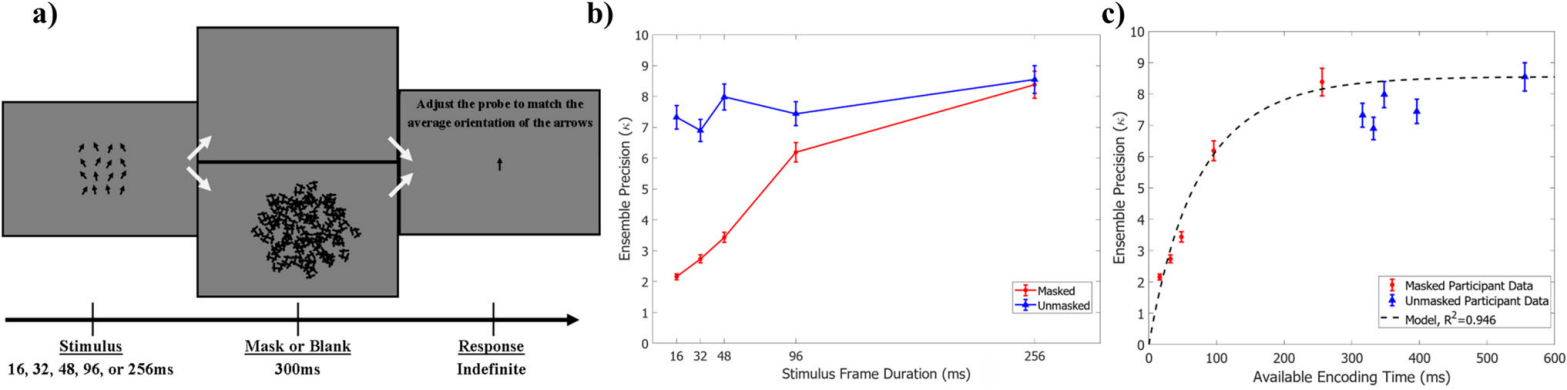
Experiment 3 trial progression and results. Panel a: An example trial progression for Experiment 3. Masking and distribution conditions varied across blocks, while the exposure duration varied within each block. Panel b: The effect of stimulus frame exposure duration and masking on the precision of orientation ensemble average reports. The precision for responses to masked trials is shown in red, while response precision for unmasked trials is shown in blue. 95% confidence intervals are shown for each precision estimate. Panel c: Participant ensemble orientation error precision plotted by available encoding time, with 95% confidence intervals displayed for each precision estimate. Model predictions (black dotted line) are an example adaptation of Eq. 1 such that $N_i$=2.27 across all available encoding durations, but the item representation precision, $\kappa _i$, is an asymptotic function of encoding time: $\kappa _{i}(t)=11.27(1-e^{-t/91})$

### Design

The experiment was organized as a 2 (masking: masked vs unmasked) x 5 (exposure duration: 16ms vs 32ms vs 48ms vs 96ms vs 256ms) x 2 (distribution: bimodal vs normal) within-participants design. Each participant completed a total of 960 trials and participated in every condition. The two distribution conditions determine the distribution of individual trial means within a block around the overall grand mean of all trials in the block. Combinations of masking and distribution conditions were split into 4 blocks of 240 trials per block, with exposure durations varying within blocks.

### Apparatus and stimuli

The apparatus and stimuli were similar to that of Experiment 2. The locations of feature values for Experiment 3 were not manipulated, and were therefore randomized. The range of orientations was restricted to $\mu \pm 24^{\circ }$ of each trial mean value. In the masked condition, a backwards pattern mask of several randomly oriented black bars that covered the full stimulus display area was used.

### Procedure

Each trial began with a centrally presented fixation cross displayed for 300ms. After a variable delay, the stimulus frame was presented for a duration corresponding to one of the exposure duration levels. After stimulus offset, either a pattern mask or a blank interval was displayed for 300ms. The response screen was then displayed, within which a probe arrow was positioned centrally on the screen (see Fig. 6a). The probe initialized at a random orientation value, and the participant would rotate the probe by moving the mouse up or down until the arrow matched the participant’s mean orientation response. Then, participants pressed the space bar to submit the response. The next trial began after response.

## Results

Participant orientation averaging performance was analyzed using a Bayesian distributional linear mixed effects model. Fixed effects for each condition, as well as all interactions, were included in the model, as were the corresponding random slopes and intercepts for estimates of the Von Mises distribution mean and precision terms. Weakly informative normal priors [$\mathcal {N}(\mu = 0,\,\sigma = 30)$] were set on all parameters.

The precision of ensemble response showed a main effect of masking, decreasing when the mask was present (estimate=-1.07, 90% CI:[-1.21 - -0.94]). Exposure duration also showed a main effect, with response precision rising as exposure duration increased (estimate=0.61, 90% CI:[0.24 - 0.97]). An interaction between masking and exposure duration was also present, such that the increase in precision with rising exposure duration was greater when a mask was present (estimate=4.78, 90% CI:[3.94 - 5.62]). A slight mean bias was present for the masking x distribution interaction (estimate = -0.03, 90% CI:[-0.06 - -0.01]), indicating that subjects may be incorporating previous trial information when the current trial information is weak. Further work will need to be done to solidify this possible memory effect. The relationship between masking and exposure duration is displayed in Fig. 6b.

As the purpose of the mask is to limit the amount of time available for information encoding into vSTM, the results in Fig. 6b can be plotted to show participant performance by the time available for encoding (Fig. 6c). The available time for encoding is equivalent to the exposure duration for masked displays, as the mask is the limiting factor in encoding time by design. For unmasked displays, subjects have an extra 300ms after the stimulus offset where encoding time is uninterrupted, after which the response screen appears. Therefore, the available encoding time for unmasked displays is plotted as the stimulus frame duration plus the extra 300ms before the response screen appears. Plotting the results in this way makes the asymptotic link between encoding time and error distribution clear.

A pattern of asymptotic growth with increasing encoding time has long been noted in the literature, particularly in describing the number of discrete items available in vSTM within masked displays of varying SOAs. In their work on information transfer, Gegenfurtner and Sperling (1993) noted a similar asymptotic relationship between encoding time and the number of discrete letters that were available in vSTM. The authors modeled the number of items available for response ($N(t))$ by a functional dependence on the capacity limit of vSTM ($N_c$), the amount of time available for encoding ($t$, in ms), and the rate of transfer ($\tau$, in ms), such that $N(t)=N_{c}(1-e^{-t/\tau })$. Contemporary theories of vSTM representation have moved towards a more continuous view of memory representations, such that a set number of items may be represented in memory with variable fidelity depending on the number of “resources” allocated towards representing each item. This gives two dimensions under which the contents of vSTM may vary over encoding times: the number of items sampled into vSTM, and the precision with which those items are represented. If ensemble summaries are generated from items being held in vSTM, the ensemble should inherit these dependencies as well.

While the participant data in Fig. 6c demonstrates the asymptotic relationship between exposure duration and performance that is typical of vSTM transfer, a question arises in how the vSTM contents may be changing to produce this pattern of behavior. Within the current theoretical framework we propose that, as long as the spatial characteristics of the stimuli are constant, limitations on encoding time adjust the representational precision of items in vSTM. Masking interrupts the transfer of item information into vSTM, creating less precise representations of the stored items. Increases in encoding time will increase information accumulation, and therefore item representation precision, to an asymptotic level.

Following this logic, we can model the relationship between ensemble response precision and available encoding time by replacing $\kappa _i$ with $\kappa _{i}(t)=K_{i}(1-e^{-t/\tau })$ in Eq. 1. We can estimate the asymptotic item precision level, $K_i$, through participant performance at the longest encoding duration window. To do so, we rearrange Eq. 1 to view the relationship between the number of items sampled ($N_i$) and the item representation precision ($\kappa _i$) needed to achieve our observed participant levels of ensemble precision:

3 $$\begin{aligned} K_i = \dfrac{\kappa _e}{1-\left( \dfrac{N_s - N_i}{N_s N_i}\right) \left( \dfrac{\kappa _e}{\kappa _s}\right) }, \end{aligned}$$

where $\kappa _e$ is the average participant ensemble precision at the longest encoding duration window, and $N_s$ and $\kappa _s$ are properties of the stimulus. For $N_i$, we use the derived value from the set size = 16 condition of Experiment 2’s to estimate an appropriate sample size value. This is an appropriate approximation, as the stimulus layout is identical in spatial characteristics between the two 16 item sets. Using Eq. 3, with $N_i$=2.27 and $\kappa _e$=8.55, we find the asymptotic item performance $K_i$=11.27. The rate parameter, $\tau$, is once again a fit parameter, with the value of best fit being $\tau$=91.

Figure 6c displays the fit of Eq. 1 to the participant data at each encoding time duration ($t$), with $\kappa _{i}(t)=11.27(1-e^{-t/91})$, and $N_i$=2.27. The model once again fits the data very well, and formalizes the relationship between ensemble performance, available encoding time, and the precision of vSTM contents.

## General discussion

In this report, we provided evidence that contradicts the perceptual account regarding ensemble representation, a view we call “Ensemble Theory”. Specifically, we have reviewed prior findings including several from our own lab, novel quantitative predictions from a classical memory architecture, and results from three new experiments that collectively demonstrate that recall of ensemble means involves operations on the contents of visual short-term memory. The results thereby contradict a host of models, both conceptual and quantitative, that assign a primary, if not sole, role to early visual cortical processes in generating ensemble representations.

In our first experiment, participants’ responses in a whole-report task were used to predict their responses in an ensemble averaging task. Specifically, data concerning the number of items recalled and the probabilities of recalling those items as a function of location on the screen were used to inform a Non-Selective Transfer model (NTM) in which a spatial attentional filter that was undirected (hence, “nonselective”), but defaulted to the center of each display, filtered a small subset of item representations from the sensory register, into vSTM. Item representations were modeled as distributions of uncertainty over a feature value axis, one distribution per item, following the Fidelity-Based Integration framework (FIM) advanced earlier by Tong and Dubé (2022, 2022b). Using the whole-report data in this way, we were able to provide an excellent account of the error distributions across 8 experimental conditions of the ensemble averaging task with the NTM using zero free parameters.

In our second experiment, we used the NTM logic to predict that the precision of participants’ ensemble mean estimates should decrease with increasing range of stimulus feature values that were within the ensembles upon which responses were to be made. This prediction was also confirmed. The results were used to fit each of the parameters of the NTM, from which remarkable consistency was found between the estimated number of items sampled and item representational fidelity estimates across conditions.

In a third experiment, we tested the prediction, again derived from NTM, that performance on an ensemble averaging task should be strongly impacted by the amount of “readout time” from iconic memory. To assess this experimentally, we varied the exposure durations of stimulus ensembles and the presence or absence of a backward pattern mask. The results were as predicted by NTM’s logical structure: unmasked performance was relatively stable and not dependent on exposure duration, while masked performance increased monotonically with performance at the longest exposure duration finally matching that of unmasked performance. We then evaluated quantitative predictions of NTM via the transfer equation derived by Gegenfurtner and Sperling (1993). Predictions of item fidelity in vSTM were generated in this way, as a function of encoding time, to simulate the error distributions at each timepoint. The model-generated data again matched human performance remarkably well.

### Relating NTM to existing models

The NTM fits most directly within the FIM framework (Tong and Dubé, 2022; Tong and Dubé, 2022b). Like FIM, NTM postulates that the ensemble arises from variably precise feature distributions. The precision of each contributing feature representation is determined by the distribution of a limited sample resource. NTM extends the FIM sampling operation by integrating an asymptotic encoding rate (Gegenfurtner and Sperling, 1993) within the model.

As currently formulated, the NTM equations may be interpreted as a *slots-plus-averaging* style of memory representation model (Zhang and Luck, 2008). A limited number of noisy item representations within vSTM contribute to the ensemble code. While the most direct interpretation, the present formalization only describes factors that contribute to the end result of the ensemble code and therefore leaves open alternative possibilities for intermediate states. Further, the current model makes no suppositions about a finite capacity limit present within working memory, which is generally a core principle of the slots-plus-averaging models. Rather, the same NTM equations would apply in a variable precision working memory framework (Bays et al., 2009; Ma et al., 2014) if items within memory are gated by a precision threshold (Schneegans et al., 2020) or *rational resource allocation* (Van den Berg and Ma, 2018). Either of these modifications to the variable precision models would therefore give stochastic capacity limits to working memory, and therefore a rationale for a limited number of items contributing to the ensemble code. The number of memory items parameter, $N_i$, may instead represent the average number of independent items that *meaningfully contribute* to the ensemble representation.

Computationally, the NTM equations are most similar to the inefficient observer models that are popular within theories of subsampled ensemble code representations (Dakin, 2001; Solomon et al., 2017; Solomon, 2021, 2010; Solomon et al., 2016). Similar to NTM, inefficient observer models assume that the distribution of errors within ensemble estimates ($\sigma ^2_{obs}$) is dependent on the spread of features present within the stimulus ($\sigma ^2_{ext}$), scaled by an estimated number of sampled items ($M$, also known as the *effective sample size*), and a later source of representational noise ($\sigma ^2_{int}$), such that: $\sigma ^2_{obs} = \sigma ^2_{int} + \dfrac{\sigma ^2_{ext}}{M}$. This model uses the standard error of the mean (SEM) to describe how sampling more items will reduce noise within the ensemble code. The SEM assumes that the sample is either drawn from a continuous population distribution, or drawn with-replacement, such that the number of sampled elements does not significantly alter the size of the population. As ensemble tasks generally use small set-sizes of items within stimuli (and therefore have small population sizes), the use of the SEM necessarily implies that samples are drawn with-replacement. This repeated sampling behavior makes the interpretation of the sample size, $M$, non-intuitive and more a measure of sampling efficiency than a number of contributing items. NTM remedies this ambiguity by applying a *finite population correction factor*, which corrects the measure to account for the hypergeometric properties of sampling without-replacement (Isserlis, 1918; Pearson, 1928). The NTM therefore provides easily interpretable estimates of the number of items contributing to the ensemble code, and provides a simple way to incorporate the stimulus set-size within calculations.

### Revisiting prior evidence for ensemble theory

In a recent, comprehensive review, Corbett et al. (2023) detail evidence for an early and “perceptual” view of ensemble representation. The authors point out two of the strongest lines of evidence in this connection: i) Studies of adaptation aftereffects in which perception of a test ensemble’s mean size is repulsed away from the mean size of a just-presented adapter cluster, ii) Studies of unilateral neglect in which ensemble averaging performance for items in the neglected visual field is spared. The other sources of evidence bearing on the question have produced a “mixed bag” of results, as the authors noted many inconsistent conclusions and confounds. These somewhat weaker lines of evidence include iii) Effects and non-effects of set size manipulations in which Ensemble Theory’s prediction of increased accuracy with set size is tested, iv) Effects and non-effects of attentional manipulations in which attempts are made to place ensembles outside of the focus of attention in order to test the prediction of sparing. This does not exhaust all possible lines of evidence for Ensemble Theory, but as far as we can tell these areas are those that have received the most attention and have attracted the greatest amount of research. We know of no stronger lines of investigation on the points of interest, and so we take each of these areas in turn.

First, consider the mean adaptation aftereffect (MAAE). The key result here is that, following the presentation of an adapter (a cluster of dots varying in diameter) judgments of the mean size of a subsequent, probe cluster are distorted away from the mean dot size of the adapter (Corbett and Melcher, 2014; Corbett et al., 2012). Corbett et al. (2023) and others interpret this effect as supporting the perceptual account. However, we find that the MAAE results do not provide definitive evidence for the perceptual account, and in fact may provide evidence against it. For one thing, the MAAE studies do not typically include any measure of performance over trials, so that it is difficult to rule out the hypothesis that such results could reflect the gradual adaptation of feature-based attentional filters to the experimental stimuli, which should produce some effect of the distribution and central tendency of those stimuli (Tong and Dubé, 2022; Tong and Dubé, 2022b).

Another, more serious, issue is due to the relatively long exposure period, the adapter stimulus information is likely to still be in short-term memory at the time of the probe onset, and prior work has already demonstrated that visual features held in short-term memory may produce repulsion in perceptual judgments of features of immediately subsequent stimuli (Kang et al., 2011). Hence, it seems possible that all that the MAAE effect tells us is that memory influences perception. This does not necessitate an early computation of summary statistical representations, and may just as well implicate the opposing view that we adopt: ensemble mean representations are computed via control operations acting on STM contents.

Our conclusion that the MAAE may actually support the NTM position follows since, if in fact the MAAE effect is an *adaptation* effect, it should only occur with viewing time sufficient to produce transfer of item information from iconic to short-term memory. Put another way, the computation of a mean occurring prior to readout (the perceptual account) should not require adaptation to produce a repulsion effect, so to the extent that the “mean adaptation after-effect” is an adaptation effect, it actually supports the NTM over the perceptual account described by Corbett et al. (2023) and others.

Second, consider the effect of set size on performance. Ensemble theories often make an assumption that all items contribute to the “global pool” (hence the term “global”), and as such, they predict that performance should generally increase with set size. Theories in which a small subset of the items contribute, as in the NTM, predict either no effect or a decline in performance with set size (depending on whether variance is held constant). As noted by Corbett et al. (2023), the results of these studies are inconsistent.

We have taken a closer look at the studies reviewed by the authors, which are detailed in Table 1. We note that two relevant datasets, those of Tong and Dubé (2022), were not included in the review and so we have included them here. One study, (Chetverikov et al., 2017), did not involve any mean estimation measures whatsoever, and so we have excluded that study. We have also excluded studies that repeated the same feature value across all items, which, taken singly, are useless for determining how many items a participant uses for estimating the mean since any number of items will yield the mean.

We note that of the 24 studies in the Table, only 5 of them show the improvement with increasing set size predicted by Ensemble Theory. Of the remainder, 12 of them show a decrease in accuracy with increases in set size and 6 show a null effect, all 18 of which are broadly consistent with the non-selective transfer model we have advanced. One study showed a non-monotonic change in accuracy which to our knowledge has not been reported in any other relevant study. Hence, we are disinclined to place much weight upon that particular finding.

We note further that, of those datasets that show a decline in performance (the hallmark of estimation via a subset of items), nearly all of them were obtained when some measure of precision was the DV. Specifically, of those 14 datasets using precision measures, 10 (> 71%) showed a decline with set size. Of the remaining 4, 3 showed no effect, and only 1 dataset showed the improvement predicted by the Ensemble theories.

Turning to those 10 remaining datasets that used a binary-response DV rather than a precision estimate, we find that the majority of those studies (i.e. 70 % of them) used percent correct (*p*(*C*)). It is of course well-known that this measure is derived from a threshold model and typically confounds changes in response bias with changes in sensitivity (Dubé, 2023; Dube and Rotello, 2012; Dube et al., 2010; Dubé et al., 2013; Dube et al., 2012; Pazzaglia et al., 2013; Rotello et al., 2015). Without recourse to further data from these studies it is difficult to tell whether accuracy is actually varying at all, or merely the willingness to endorse a particular response. Some of these datasets used a 2AFC task, long believed to be “immune” from such issues although even this assumption has been shown to be erroneous on several grounds (Jou et al., 2016; Starns et al., 2017). Given all of these problems with the DVs of these studies, it is perhaps no surprise that most of the inconsistency remarked upon by Corbett et al. (2023) stems from these same studies: 3 null effects, 2 declines, 4 improvements, and one non-monotonic result were observed when *p*(*C*) was used. The only one of these studies that included both *hits* and *false alarms* (and a signal detection analysis, albeit an equal-variance one) produced one of the 3 null results.

**Fig. 7:**
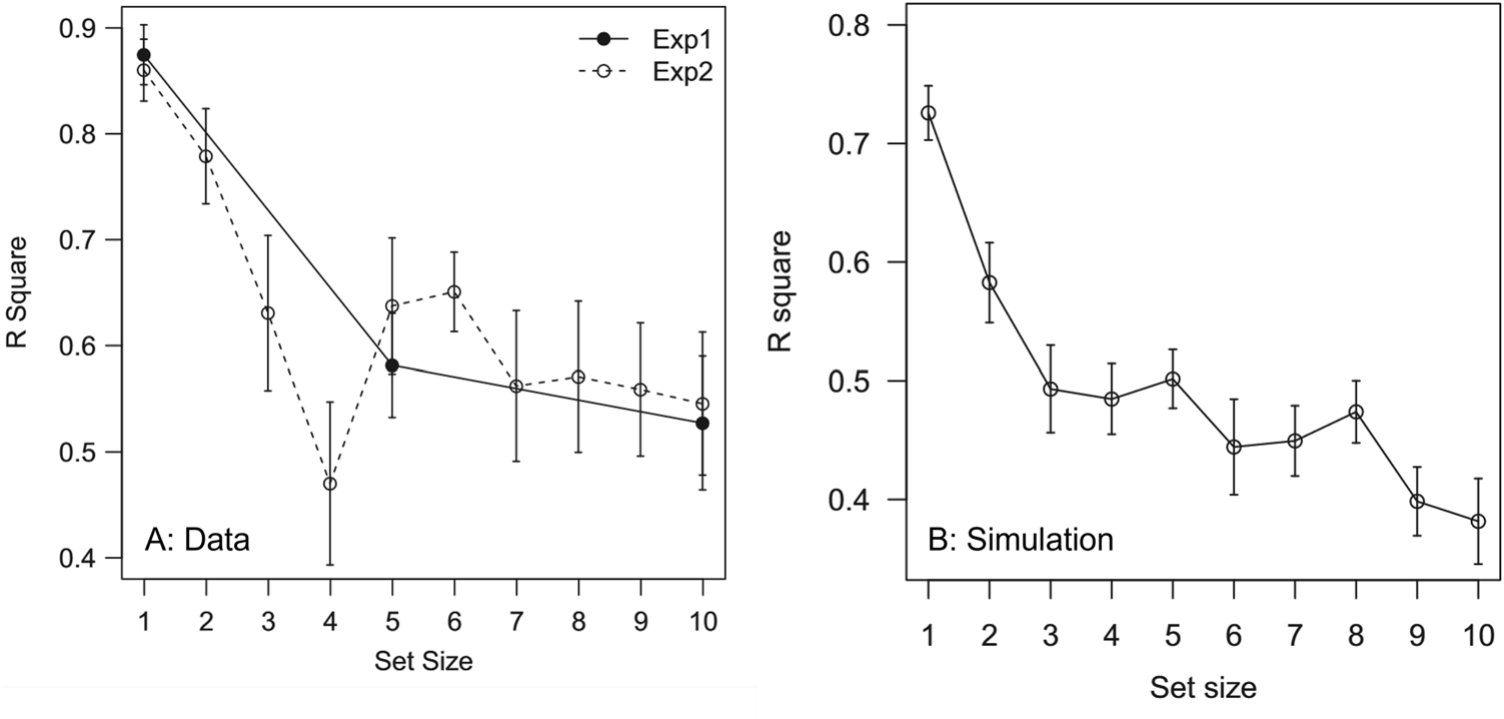
Data from Tong and Dubé (2022) demonstrating nonlinear decrease in mean estimation accuracy as a function of set size (**A**) and simulation using the Fidelity-Based Integration model (**B**)

Notice that the study by Tong and Dubé (2022) is unique in including both the method of reproduction and a very large range of set sizes. These data, plotted in Panel A of Fig. 7, show a decrease in precision of participants’ mean estimations which is nonlinear: Very large drops in precision across set sizes below the putative STM capacity of 4 items are followed by very small changes at larger set sizes. This is consistent with our non-selective transfer model in that it inherits the FIM assumption that both slot-like capacities and informational resolution limits are gradually taxed such that diminishing returns beyond slot capacity reflect primarily the limiting process of resource spreading. To verify this, we include the simulations from the FIM core of the transfer model, in Panel B of the figure.

Looking more closely at Table 1, we find that of the 6 null effects that have been reported, 4 of them fail to include any set size below 4 items. This same limitation holds for our own data in Experiment 2 of this report. Since, as shown in Fig. 5, our model can predict null effects in performance above item limits, we find that the majority of these null effects are also consistent with our position. Furthermore, we note that even the lone non-monotonicity in the data in Table 1 could in principle be accounted for without Ensemble Theory: both the data, and the FIM model, produce a non-monotonicity in precision of participants’ mean estimates above set sizes of 4 unique items.

**Table 1:** Compilation of reported set size effects from ensemble coding studies

Experiment	Set Size	Feature	Task	DV	Effect
Corbett and Oriet (2011) E1	5–11	Circle Size	Member ID, prototype lures	Accuracy: p(C), HR, FAR	Null
Maule and Franklin (2015) E1	4, 8, 16	Circle hue	2AFC: Which matches mean?	Accuracy: p(C); Latency: Mean Correct RT	Decline
Maule and Franklin (2015) E2a, 12JND	2, 4	Circle hue	2AFC: Which matches mean?	Accuracy: p(C); Latency: Mean Correct RT	Decline
Maule and Franklin (2015) E2a, 20JND	2, 4	Circle hue	2AFC: Which matches mean?	Accuracy: p(C); Latency: Mean Correct RT	Null
Maule and Franklin (2015) E2a, 28JND	2, 4	Circle hue	2AFC: Which matches mean?	Accuracy: p(C); Latency: Mean Correct RT	Improvement
Robitaille and Harris (2011) E1	2–12	Circle Size	Is target < mean?	Accuracy: p(C); Latency: Mean Correct RT	Improvement
Robitaille and Harris (2011) E2	2–12	Line tilt	Is target < mean?	Accuracy: p(C); Latency: Mean Correct RT	Null (Accuracy)
Brezis et al. (2015) E1/E2	4, 8, 16	Numerals	Is mean > 50?	Accuracy: RMSD; Latency: Median RT	Nonmonotonic (Accuracy)
Brezis et al. (2015) E3	4, 8, 16	Numerals	Is mean > 50?	Accuracy: RMSD; Latency: Median RT	Improvement
Brezis et al. (2015) E4	4, 8, 16	Numerals	Is mean > 50?	Accuracy: RMSD; Latency: Median RT	Improvement
Ji and Pourtois (2018) E1	4, 8, 16	Face emotion	Visual Analogue Rating Slide	Precision: (observed – predicted rating)	Decline
Ji and Pourtois (2018) E2	4, 8, 16	Face emotion	Visual Analogue Rating Slide	Precision: (observed – predicted rating)	Decline
Ji and Pourtois (2018) E3	4, 8, 16	Face emotion	Visual Analogue Rating Slide	Precision: (observed – predicted rating)	Null
Marchant et al. (2013) E1	4, 8	Circle Size	Reproduction task	Precision: Absolute Error	Decline
Marchant et al. (2013) E2	4, 8, 16	Circle Size	Reproduction task	Precision: Absolute Error	Decline
Allik et al. (2013) E1	1, 2, 4, 8	Circle Size	Is target < mean?	Precision: PMF Slope	Null
Allik et al. (2013) E2, Ref = 300	1, 4	Circle Size	Is target < mean?	Precision: PMF Slope	Decline
Allik et al. (2013) E2, Ref = 150	1, 4	Circle Size	Is target < mean?	Precision: PMF Slope	Decline
Allik et al. (2013) E2, Ref = 75	1, 4	Circle Size	Is target < mean?	Precision: PMF Slope	Decline
Tong and Dubé (2022) E1	1, 5, 10	Line length	Reproduction task	Precision: $R^2$	Nonlinear decline
Tong and Dubé (2022) E2	1–10	Line length	Reproduction task	Precision: $R^2$	Nonlinear decline
Utochkin and Tiurina (2014) E1	4, 8, 16	Circle Size	4AFC	Pseudoprecision (see text)	Decline
Utochkin and Tiurina (2014) E2	4, 8, 16	Circle Size	4AFC	Pseudoprecision (see text)	Null
Utochkin and Tiurina (2014) E3	4, 8, 16	Circle Size	4AFC	Pseudoprecision (see text)	Nonlinear improvement

Third, consider studies of ensemble averaging in the absence of item selection. Of these, Corbett et al. (2023) note a large amount of inconsistency. The strongest line of evidence appears to come from studies of unilateral spatial neglect (Pavlovskaya et al., 2015; Yamanashi Leib et al., 2012). In these studies, it is typically shown that when ensembles are presented to a neglected hemifield, the patient is still accurate in estimating ensemble means. The studies all suffer from the same confound, however, which is that the patients are not completely unable to recall individual items from the neglected field of vision. Since, from the current work and prior literature on subsampling, one can produce typical ensemble mean performance with only a few items, it follows that USN patients, who can still recall at least 1 or 2 items from the neglected field, would be spared in their ensemble mean judgments.

### Centroid processing

A number of rigorous, quantitative and empirical studies have been reported by Sperling, Chubb, and colleagues that strongly suggest rapid computation of centroids of spatially-intermingled dots varying in some identifying feature (Drew et al., 2010; Inverso et al., 2016; Rodriguez-Cintron et al., 2019; Sun et al., 2016b, a, 2018). In the most recent such study, Gan et al. (2023) write:

Human visual system can extract summary statistical information from groups of similar items in a brief glance. Such ensemble statistics are interesting because they result from brain mechanisms that can quickly distill a large amount of sensory information for subsequent cognitive processes that have much lower capacity [...] Humans are also capable of selectively forming statistics out of spatially intermingled visual stimuli defined by different features.

This reflects the unwarranted influence of the perceptual account or Ensemble Theory: The story is that Ensemble means are computed rapidly by neural pooling prior to transferring the resulting mean to STM. The 2D centroid of a particular, briefly-presented cloud of dots is an example of such an ensemble representation. As in other centroid estimation papers, the authors then proceed to use variations of the centroid estimation task to estimate the parameters of spatial attentional filters, referred to by Koch and Ullman (1985) as salience maps. Using these methods, Gan et al. (2023) derive evidence for the concurrent deployment of multiple such maps within the observer.

While we find the studies by Sperling and colleagues to be persuasive and elegant in many respects, we do not find any convincing evidence for Ensemble Theory in these studies. First, in none of the Sperling group’s papers is there any case in which a control condition is included that requires responses to a position other than the centroid. As such, the conclusion that centroids are represented as in Ensemble Theory does not necessarily follow, and furthermore the centroid phenomena are readily explained by classic findings regarding spatial location.

For instance, Sperling (1960) established, among other things, that participants’ abilities to locate and report a single item from a particular spatial location in a briefly-presented array are remarkably good when a pre-cue, concurrent cue, or near-immediate post-cue is given. Notice the correspondence with the basic centroid setup, in which the participant does not even have to report an item from a location, but merely report a location.

The findings from multiple, concurrent centroid judgments, which the Sperling group appears to interpret as even stronger evidence for specialized ensemble mean processing, are in reality strongly implied by what we already know, since one can define the locations within the cells of any $m{\times }n$ array in terms of the *mn* “centroids” by a mere change of notation, in which case the location of any point on the screen can be defined as a “centroid” with respect to the local frame of reference. It is clear that the distinction between centroid processing and location processing is, at best, highly questionable.

Put simply, until it is demonstrated that centroid locations are processed in some way that differentiates them from any other locations in a given dot cloud, we have no reason to conclude that anything has been discovered about centroids that was not already discovered about any location in general, in Sperling’s own prior work.

At the same time, the conclusion of whether or not centroid estimation is an example of an ensemble averaging process appears to have little to no bearing on the main findings of Sperling’s group, since the modeling and experiments are not typically directed toward the understanding of ensemble representations but rather are *piggy-backing* on the phenomenon of rapid, efficient processing of a particular location (2D centroid) in order to learn something about spatial attention filters. These latter are the foci of the mathematical models advanced by Sperling and colleagues; ensemble representation appears to be a non-essential construct in these studies that is merely along for the ride.

### Obligatory averaging and veiled control

As we noted in the *Introduction*, Dubé and Sekuler (2015) concluded a review of the ensemble representation literature by positing that i) ensemble averages are computed according to the perceptual account and ii) they are computed obligatorily. The current work suggests that i) is incorrect, but from what we have established thus far, it does not follow that ii) is in error. This is because the claim of obligatory averaging was based on the fact that the means of ensembles of stimuli appear to influence or bias responding in tasks that have no requirement to represent or estimate a mean. These findings do not necessarily confirm or negate the use of control processes, and neither does the use of control processes confirm or negate the claim that averaging is obligatory.

Specifically, the literature regarding controlled processing of STM and LTM representations shows that such processing need not be available to conscious awareness. Implicit or unconscious control processes are referred to as *veiled control processes* (Shiffrin and Schneider, 1977). For this reason, the fact that effects of ensemble means have been observed both in explicit tasks like mean estimation, as well as implicitly in tasks with no requirement to represent a mean, does not have any bearing *à priori* on the logic we have used to derive our conclusions. In fact, Tong and Dubé (2022b) have demonstrated that the same basic FIM architecture can account for both the explicit ensemble representation tasks and the implicit, biasing effects of ensembles’ means on single-item judgments and reproductions.

### Limitations and future directions

We cannot rule out the possibility that an Ensemble account might be proposed to explain our results. For instance, considering the results of Experiment 3, one might imagine an account in which ensemble codes are constructed via the usual mechanisms of global pooling or distributed attentional states over items registered by sensory and perceptual mechanisms. The ensemble representation is then transferred to a short-term memory store. However, when encoding time is restricted, this transfer of the pre-computed ensemble code is likewise restricted, resulting in a mixture of trials in which participants have varying degrees of access to the ensemble representation.

Our results from Experiment 3 do not seem capable of distinguishing between such an account and the one we offer, and we think this Ensemble account would not be easy to falsify with manipulations targeting encoding restrictions or load. However, taken as a whole, our results across all three experiments argue against such an account. Specifically, the Ensemble account would also have to explain the close correspondence between the memory and ensemble averaging data of our first experiment as well as the range effects of the second. Regarding the former, such an account would be forced into model mimicry since collectively the three experiments consistently exhibit the classical hallmarks of memory systems, such as negatively-accelerated performance curves over encoding time and 4-item limits, the latter of which were derived from a memory task differing only in the response requirement and which predicted ensemble averaging data from the same participants.

It may also be informative to consider other manipulations for testing the NTM and competing accounts. One possibility is to use a dual-task interference design. However, we do not think such an approach would be very useful for distinguishing between the perceptual and the memory views. Specifically, in order to carry out the typical ensemble perception task, the participant must remember the mean for long enough to respond to the subsequent probe. Hence, any modality-specific interference from a dual task could impact performance through interference with the memory representation of the ensemble regardless of whether the representation was formed via control processes or was formed via “early” or perceptual processes and then transferred to STM (as in the model described above). Similarly, the absence of any such effect could be read as supportive of a perceptual account such as one in which the ensemble representation, being akin to a single “item” with respect to memory storage, would presumably require fewer resources to maintain than a set of representations in STM. It is for reasons like these that we used other classical manipulations for taxing STM resources in our current work (encoding time) as well as direct within-subject comparisons of ensemble averaging and iconic memory transfer.

Further, while we have provided a set of experimental results to demonstrate the reasoning behind the NTM, the list of accounted for phenomena is less than comprehensive. Future work is necessary to extend the NTM to account for the ensemble code’s role in findings of outlier exclusion (Haberman and Whitney, 2010; Epstein et al., 2020), the representation of skewed distributions (Iakovlev and Utochkin, 2023), and the rapid categorization of visual information (Khvostov et al., 2021), among others. However, we believe a major goal of theory is not only to explain new and existing data, as we do here, but also to inspire new experimental tests for future work to move the literature forward. We hope that our proposal will motivate such endeavors.

## Conclusions: Playing 20 questions with nature

In his classic 1973 paper, Allen Newell voiced concerns about the proliferation of incremental experiments surrounding new phenomena. One of the concerns he raised was that the absence of a full processing model for much of the work under discussion served to encourage a rapidly ballooning, but possibly directionless, literature. We find that his criticism applies in many ways to the literature today, and in particular the ensemble representation literature. We have adopted a view that, while not comprehensive, at least moves beyond the disembodied processing loci of current theories. That is, we adopted a classical memory processing model to direct our experimental work, and formalized key aspects, such as information transfer rate between memory stores, that were most useful for testing theories of ensemble representation.

Our research has led us to view the transfer of ensemble-relevant information between early sensory-perceptual space and vSTM space as akin to Descartes’ famous example of a cane-wielding peripatetic. The construction of representations of novel stimuli involves a process of sampling and recovery (Shiffrin and Steyvers, 1997) at a sub-symbolic level, where the sub-symbolic “stuff” is the evidence over a feature value, the sampling process is the retrieval or transfer of the evidence-feature mapping into an activated state or participant to a process called vSTM (depending on one’s conceptualization of vSTM as an activated subset vs. a module), and recovery involves something equivalent (but not identical) to a statistical inference. In short, the evidence we have presented supports an Approximation Theory for understanding ensemble representations, in which such representations are constructed via (possibly veiled) control processes acting to integrate information maintained in vSTM.

Since i) no averaging (weighted, arithmetic, geometric, or otherwise) occurs in the NTM model, ii) ensemble representations are generated from STM contents in the NTM model, and since iii) the NTM and FIM models provide excellent descriptions and predictions of prior data, and the NTM’s predictions were also supported in the new data we report, we conclude that the perceptual average in ensemble coding is neither perceptual nor an average.

## Data Availability

All data and experimental materials are available from the authors upon reasonable request.

## Appendix

### Extended model discussion

The current formalization of the NTM provided in Eq. 1 (reprinted here from the main text) is a simple demonstration of the underlying theory.

4 $$\begin{aligned} \kappa _e = \dfrac{\kappa _i}{\left( \dfrac{N_s - N_i}{N_s N_i}\right) \left( \dfrac{\kappa _i}{\kappa _s}\right) +1} \end{aligned}$$

The equations relate closely to the *noisy observer models* (Solomon, 2010; Solomon et al., 2017; Dakin, 2001; Solomon et al., 2016). This becomes more clear if an approximation is made between the circular precision ($\kappa$) and the variance ($\sigma ^2$), such that: $\sigma ^2 \approx \dfrac{1}{\kappa }$. Applying the variance approximation in Eq. 1 gives:

5 $$\begin{aligned} \dfrac{1}{\sigma ^2_e} = \dfrac{\left( \dfrac{1}{\sigma ^2_i}\right) }{\left( \dfrac{N_s - N_i}{N_s N_i}\right) \left( \dfrac{\sigma ^2_s}{\sigma ^2_i}\right) +1} \end{aligned}$$

which simplifies to:

6 $$\begin{aligned} \sigma ^2_e = \sigma ^2_i + \left( \dfrac{N_s-N_i}{N_sN_i}\right) \sigma ^2_s \end{aligned}$$

where $\sigma ^2_e$ is the variance of ensemble average response errors, $\sigma ^2_i$ is the average noise present in the representation of sampled items, $N_s$ represents the number of items present in the stimulus and therefore within sensory memory, $N_i$ represents the number of encoded items that contribute to the ensemble representation, and $\sigma ^2_s$ is the spread of feature values present in the stimulus and therefore sensory memory.

The main computational distinction between Eq. 3 and previous noisy observer type models is the use of the *finite population correction* (FPC) factor: $\dfrac{N_s-N_i}{N_sN_i}$. The use of this correction factor scales the noise due to sampling relative to the proportion of the complete population that has been sampled (Isserlis, 1918; Pearson, 1928). For example, if all items from the stimulus are sampled, no noise from the stimulus should contribute to the ensemble code as the complete population space has been sampled. Within the context of the current model, the multiplication of the FPC factor and the variance of stimulus feature values describes the contribution of variation in the sampled item distribution means to the ensemble estimate.

## Footnotes

1 Note: $\kappa$ is used as the circular precision estimate of the Von Mises distribution for all analyses and subject results. For modeling, $\kappa$ is approximated as $\kappa \ \approx 1/\sigma ^2$ for computational simplicity.

## Declarations

### Code availability

All experiment and analysis code is available from the authors upon reasonable request.
